# Artificial intelligence-assisted endoscopic ultrasonography for the detection of a small T1 pancreatic ductal adenocarcinoma in a difficult-to-puncture location

**DOI:** 10.1055/a-2944-3582

**Published:** 2026-09-10

**Authors:** Yukitoshi Matsunami, Shuntaro Mukai, Hirohito Minami, Kazuki Hama, Takao Itoi

**Affiliations:** 1 Department of Gastroenterology and Hepatology 13112 Tokyo Medical UniversityShinjuku-kuTokyoJapan

**Fig. 1:**
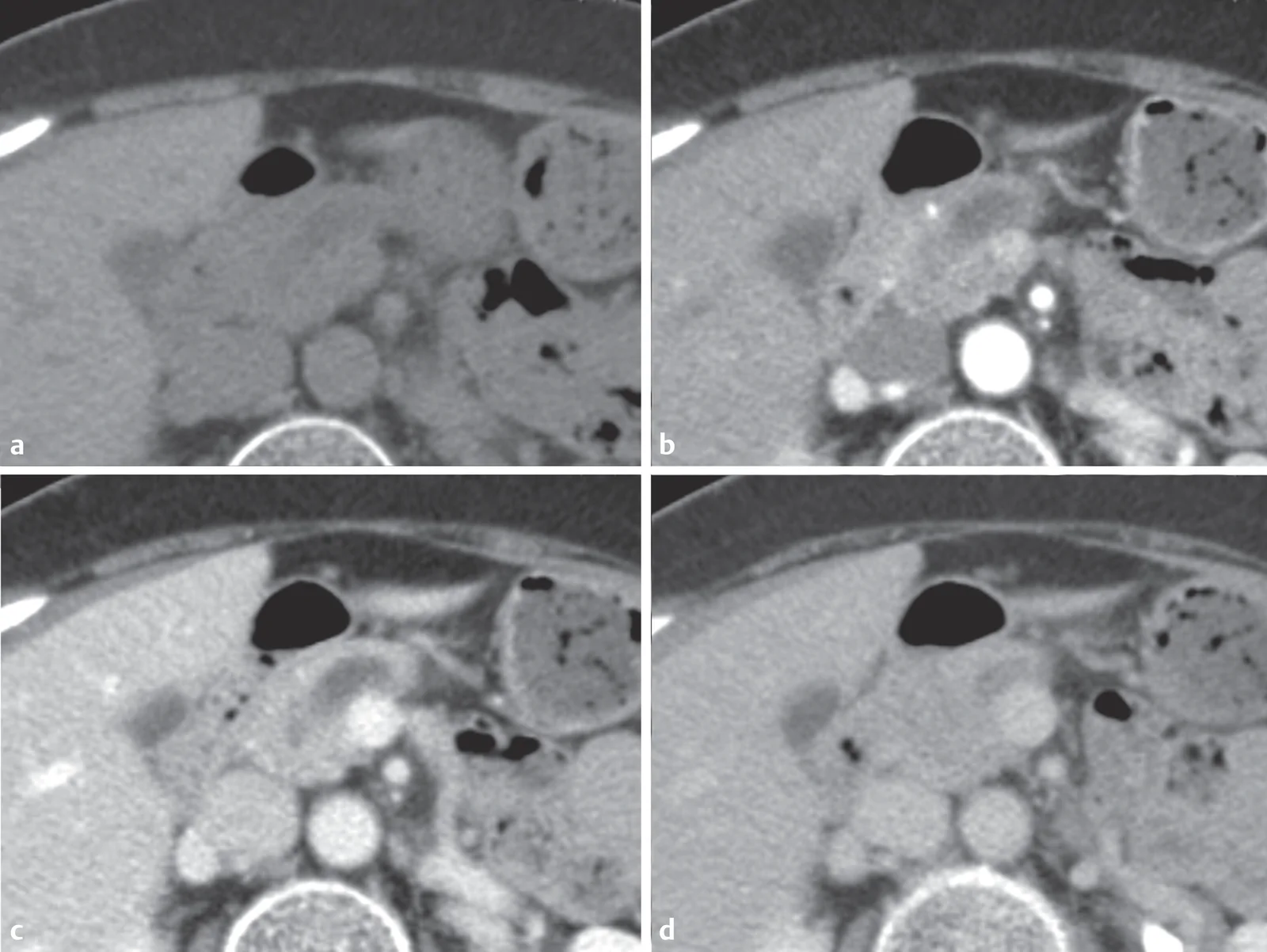
Dynamic CT showing the dilation of the MPD without an identifiable pancreatic mass. ( **a** ) Plain CT. ( **b** ) Artery phase. ( **c** ) Portal phase. ( **d** ) Delay phase.

**Fig. 2:**
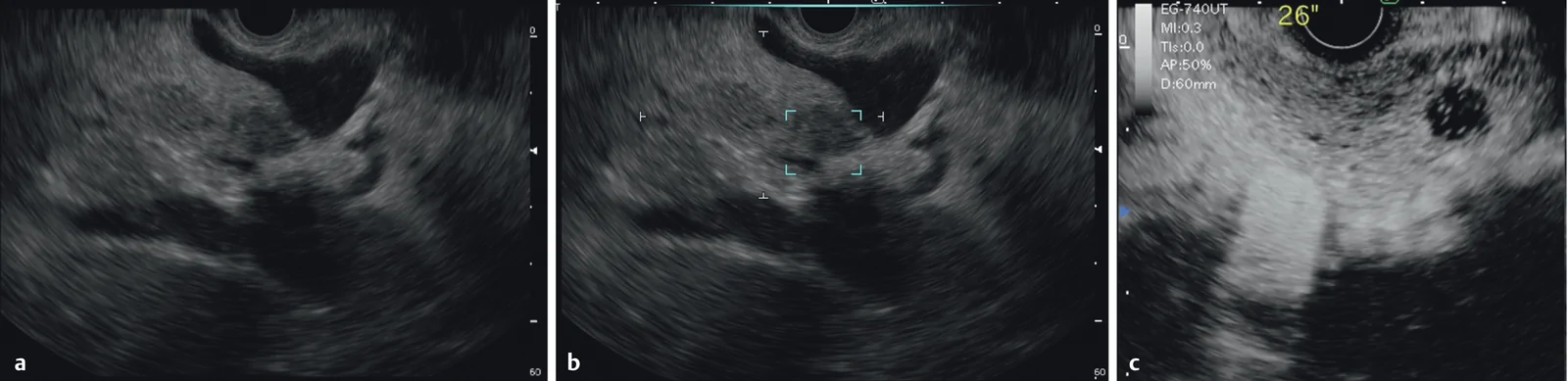
EUS findings of the pancreatic lesion. ( **a** ) Conventional B-mode EUS showing a 10-mm hypoechoic lesion in the pancreatic head. ( **b** ) Real-time AI-assisted EUS image of the same section as panel a. The pancreatic parenchyma and solid lesion are enclosed by white and light blue bounding boxes, respectively. ( **c** ) CE-EUS demonstrating a hypovascular lesion.

**Fig. 3:**
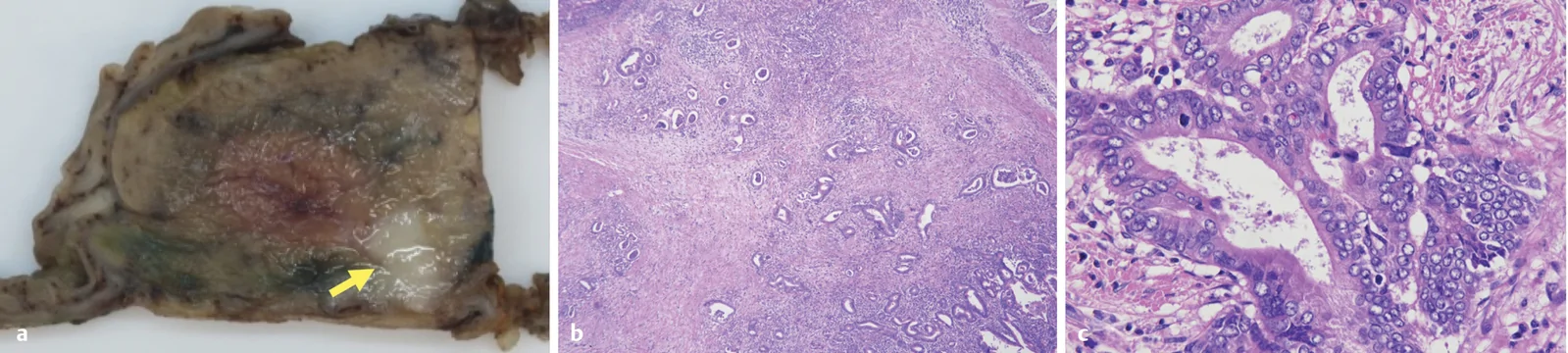
Histopathological examination confirmed stage IA (T1N0M0) PDAC. ( **a** ) A macroscopic view of the resected specimen. The yellow arrow indicates the tumor. ( **b** ) Low power H&E staining demonstrating invasive gland-forming adenocarcinoma with prominent desmoplastic stroma. ( **c** ) High power H&E staining demonstrating irregular atypical glandular proliferation, consistent with PDAC.

Endoscopic ultrasonography (EUS) is the most sensitive imaging modality for detecting small pancreatic lesions. However, the identification of small pancreatic ductal adenocarcinoma (PDAC) remains challenging and highly operator dependent. 1​ Recently, an artificial intelligence (AI)-assisted EUS system, EW10-US01 (CAD EYE; FUJIFILM Corporation, Tokyo, Japan), has become commercially available in Japan. 2​ This real-time AI-assisted software analyzes EUS images to identify pancreatic parenchyma and highlight areas suspicious for solid pancreatic lesions using visual alerts. We report a case in which this AI-assisted EUS system supported the detection of a small T1 PDAC in a difficult-to-puncture location that had not been identified on dynamic computed tomography (CT). A 60-year-old woman was referred to our institution for the evaluation of main pancreatic duct (MPD) dilation. Dynamic CT demonstrated the dilation of the MPD without an obvious pancreatic mass ( Fig. 1a–d ). EUS revealed a 10-mm hypoechoic lesion in the pancreatic head, which was readily identified and simultaneously highlighted using the AI-assisted detection system. Contrast-enhanced EUS demonstrated a hypovascular pattern ( Fig. 2a–c , Video 1 ). EUS-guided tissue acquisition (EUS-TA) was attempted; however, safe needle puncture was difficult because of intervening vessels from both the duodenal bulb and the second part of the duodenum. Therefore, based on the overall clinical and imaging findings, the lesion was considered highly suspicious for PDAC, and pancreaticoduodenectomy was performed. Histopathological examination confirmed stage IA (T1N0M0) PDAC, and curative resection was achieved ( Fig. 3a–c ). This case highlights the potential utility of real-time AI-assisted EUS for supporting the detection of small pancreatic lesions, particularly in technically challenging situations in which EUS-TA may be difficult to perform safely. 3​

**Video 1** Real-time AI-assisted recognition of pancreatic parenchyma and the detection of a small solid pancreatic lesion during EUS. EUS-guided tissue acquisition (EUS-TA) was technically difficult because of the intervening vessels.


Endoscopy_UCTN_Code_TTT_1AS_2AD
